# Metformin therapy modifies corneal neuroimmune abnormalities in people with type 2 diabetes

**DOI:** 10.1007/s00125-025-06399-2

**Published:** 2025-03-07

**Authors:** Amy T. Tsoi, Janice Sng, Shyam S. Tummanapalli, Tushar Issar, Ann M. Poynten, Kerry-Lee Milner, Maria Markoulli, Roshan Dhanapalaratnam, Arun V. Krishnan

**Affiliations:** 1 School of Clinical Medicine, University of New South Wales, Sydney, NSW Australia; 2 School of Optometry & Vision Science, University of New South Wales, Sydney, NSW Australia; 3 Department of Endocrinology, Prince of Wales Hospital, Sydney, NSW Australia; 4 Department of Neurology, Prince of Wales Hospital, Sydney, NSW Australia

**Keywords:** Corneal nerve injury, Diabetic peripheral neuropathy, In vivo corneal confocal microscopy, Metformin

## Abstract

**Aims/hypothesis:**

Diabetic peripheral neuropathy is a debilitating microvascular complication of diabetes mellitus, with limited disease-modifying therapies to date. This study aimed to assess the effect of metformin on the corneal sub-basal nerve plexus as a peripheral neuropathy outcome measure in people with type 2 diabetes.

**Methods:**

A cohort of 36 participants with type 2 diabetes receiving metformin therapy were recruited and underwent clinical assessment, corneal confocal microscopy and nerve conduction studies. Concurrently, 36 participants with type 2 diabetes not receiving metformin therapy were selected as disease controls and matched to participants on metformin therapy for age, sex, diabetes duration, BMI, eGFR, HbA_1c_, use of other oral glucose-lowering agents and therapies used for the treatment of the metabolic syndrome. Additionally, 25 healthy control participants were assessed and matched for age and sex. Medical record data over the previous 20 years were analysed for prior and current metformin use in all participants with type 2 diabetes.

**Results:**

Participants receiving metformin therapy had higher corneal nerve fibre density (*p*=0.020), corneal nerve fibre length (*p*=0.020) and corneal fractal dimension (*p*=0.003) compared with those not receiving metformin therapy. The inferior whorl dendritic cell density was significantly lower in the metformin group compared with the non-metformin group (*p*=0.043).

**Conclusions/interpretation:**

Metformin treatment is associated with superior corneal nerve parameters and neuroimmune tone in the corneal sub-basal nerve plexus. This study provides further evidence that metformin may be neuroprotective in diabetic peripheral neuropathy.

**Graphical Abstract:**

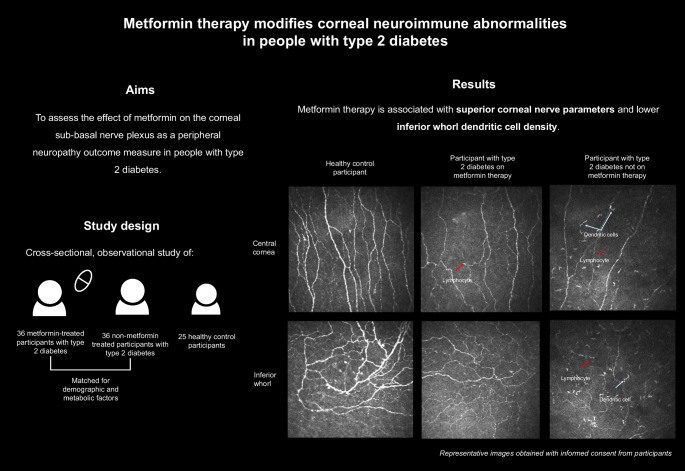


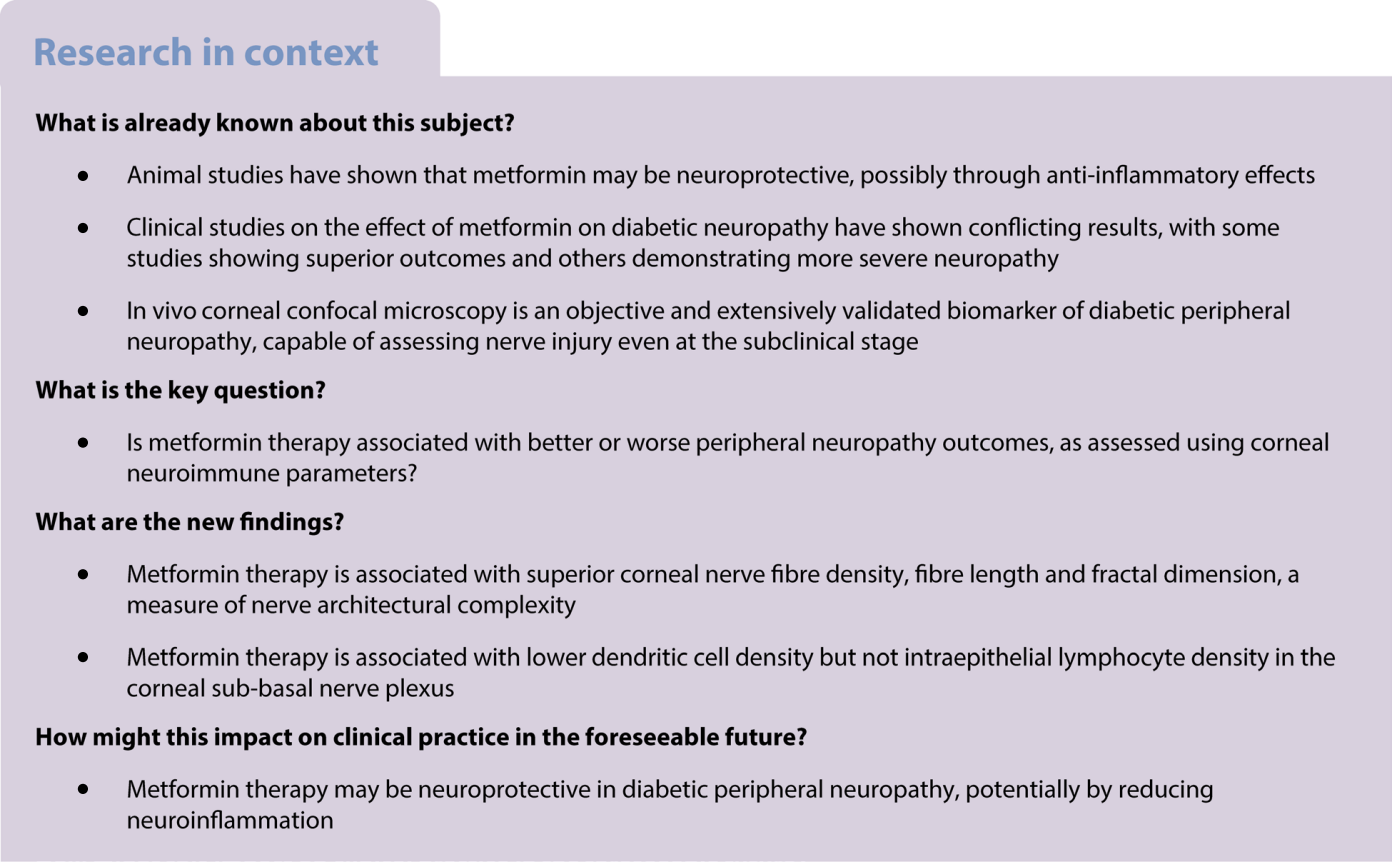


## Introduction

Diabetic peripheral neuropathy (DPN) affects 50% of people with type 2 diabetes [1]. Metformin is the most commonly prescribed oral glucose-lowering agent for the treatment of type 2 diabetes. In the cornea, diabetes-induced denervation reduces the regenerative capacity of epithelial cells to recover from injury. Diabetic corneal neuropathy, along with other pathophysiological mechanisms, such as pyroptosis, oxidative stress and fibrogenesis, contribute to the development of diabetic neurotrophic keratopathy, a complication that carries the potential for irreversible vision loss [2]. Corneal nerve parameters measured with in vivo corneal confocal microscopy (IVCCM) are objective, validated biomarkers of DPN, capable of assessing nerve injury at the subclinical stage, where interventions are most likely to be effective [3]. Since protection against neuroinflammation has been posited as a potential mechanism by which metformin exerts its neurotrophic effects in animal studies [4], IVCCM may be useful in exploring these findings in humans as it allows for the quantification of corneal immune cell populations. This study therefore aims to explore the impact of metformin on corneal neuroimmune parameters using IVCCM.

## Methods

### Participant selection

One hundred and forty-three participants with type 2 diabetes were consecutively recruited and screened from a tertiary referral diabetes centre. Thirty-six participants receiving metformin were matched to 36 participants who had not received metformin in the 6 months prior to obtaining consent. The participant’s sex was determined by self-report and cross-referenced with information in their medical record. As participants were consecutively recruited, their ethnicity, religion and socioeconomic status were not used to determine selection. Groups were matched for age, sex, diabetes duration, BMI, HbA_1c_, eGFR, systolic BP, serum lipids and use of antihypertensive therapies, lipid-lowering agents and other oral glucose-lowering agents. Twenty-five age- and sex-matched control participants were also recruited. Studies were conducted with informed consent and institutional ethics approval (Human Research Ethics Committee of the South Eastern Sydney Local Health District HREC Ref. 14/012). Exclusion criteria were age <18 years; history of peripheral neuropathy unrelated to diabetes; current ocular infection; corneal ectasia; corneal abrasion and contact lens use; history of refractive surgery; anterior segment trauma; cataract surgery in the last 6 months; and use of any ocular medications Schedule 3 or above, including topical ocular corticosteroids. Exclusion criteria for control participants included diagnosis of diabetes; current history of central or peripheral nervous system disease; BMI >27 kg/m^2^; and use of medications for hypertension or hyperlipidaemia.

### Assessments

IVCCM (Heidelberg Retinal Tomograph III Rostock Cornea Module, Heidelberg Engineering, Heidelberg, Germany) and quantification of nerve parameters were performed using validated software (ACCMetrics, University of Manchester, Manchester, UK), as per standard protocols [5]. Corneal dendritic cells and intraepithelial lymphocytes (IEL) were independently counted by two observers blinded to the participant’s treatment, using ImageJ Software (National Institutes of Health, Bethesda, MD, USA), as per previously established definitions based on end-to-end lengths and presence of dendrites [6]. DPN severity was calculated using the Total Neuropathy Score (TNS), which incorporates symptoms and signs with nerve conduction study parameters [7]. Data from hospital electronic and hardcopy records over the past 20 years were used to record prior and current metformin use in all participants with type 2 diabetes.

### Sample size calculation

Sample size calculation was performed using G* Power 3.1.9.6 (Heinrich Heine University, Dusseldorf, Germany), based on corneal nerve fibre length in participants with diabetes and DPN (12.33 ± 2.66 no/mm^2^) compared to participants with diabetes without DPN (14.88 ± 1.91 no/mm^2^) [8]. A minimum sample size of 14 participants in each group was adequate to detect a difference with 80% power at an alpha level of 0.05.

### Statistical analysis

Statistical analysis was performed using SPSS version 27.0 (IBM Corp, Armonk, NY, USA). The normality of continuous data was determined using the Shapiro–Wilk test. Categorical data were analysed using the χ^2^ test. ANOVA, Kruskal–Wallis tests, independent *t* tests and Mann–Whitney *U* tests were used to analyse continuous data, with post hoc Bonferroni corrections applied where appropriate to correct for multiple testing. Values are presented as mean ± SE. A *p* value of <0.05 was regarded as statistically significant. Multiple regression analysis was undertaken to assess the influence of possible confounders, including age, disease duration, HbA_1c_, eGFR and insulin use.

## Results

Participant demographics are summarised in Table 1. In the metformin group, the mean duration of metformin therapy was 14.9±3.0 years, and the mean daily metformin dose was 1295±83 mg. In the non-metformin group, 16 participants never received metformin. For the remaining 20 participants, the mean duration of metformin treatment was 12.7±1.5 years and the mean time since cessation was 5.0±0.8 years.

**Table 1:** Participant demographics

Variable	On metformin (*n*=36)	Not on metformin (*n*=36)	Control participants (*n*=25)	*p* value (metformin and non-metformin)	*p* value (metformin and controls)
Age (years)	64.5±2.1	65.3±1.7	65.4±1.2	0.773	0.732
Sex (M/F)	23/13	22/14	14/11	1.000	0.535
Diabetes duration (years)	15.4±1.4	17.2±2.0		0.458	
BMI (kg/m^2^)	29.2±0.8	30.6±1.0	25.2±1.0	0.398	0.002**
HbA_1c_ (mmol/mol)	69.5±3.0	75.1±3.5		0.193	
HbA_1c_ (%)	8.5±0.3	9.0±0.3		0.193	
eGFR (ml/min per 1.73m^2^)	67.2±21.8	62.2±24.6		0.441	
CKD (%)	31	47		0.227	
Mean SBP (mmHg)	133.0±2.7	133.6±2.5		0.162	
Triacylglycerol (mmol/l)	2.1±0.3	1.9±0.2		0.957	
HDL-cholesterol (mmol/l)	1.2±0.1	1.2±0.1		0.650	
LDL-cholesterol (mmol/l)	1.9±0.2	2.4±0.2		0.252	
On DPP4i (%)	47	44		1.000	
On sulfonylurea (%)	14	19		0.527	
On SGLT2i (%)	39	22		0.125	
On GLP-1RA (%)	6	8		1.000	
Insulin use (%)	58	86		0.009**	
On antihypertensives (%)	77	69		0.422	
On lipid-lowering agents (%)	89	75		0.126	
On aspirin (%)	33	28		0.262	
TNS	4.6±1.0	7.1±1.3		0.175	
Sural amplitude (µV)	9.5±1.2	7.6±1.2		0.240	
Tibial amplitude (mV)	6.9±0.6	5.6±0.8		0.160	

Data are presented as mean ± SE or % unless otherwise stated

^**^p<0.01

CKD, chronic kidney disease; DPP4i, dipeptidyl peptidase-4 inhibitor; F, female; GLP-1RA, glucagon-like peptide-1 receptor agonist; M, male; SBP, systolic blood pressure; SGLT2i, sodium–glucose cotransporter 2 inhibitor

**Fig. 1:**
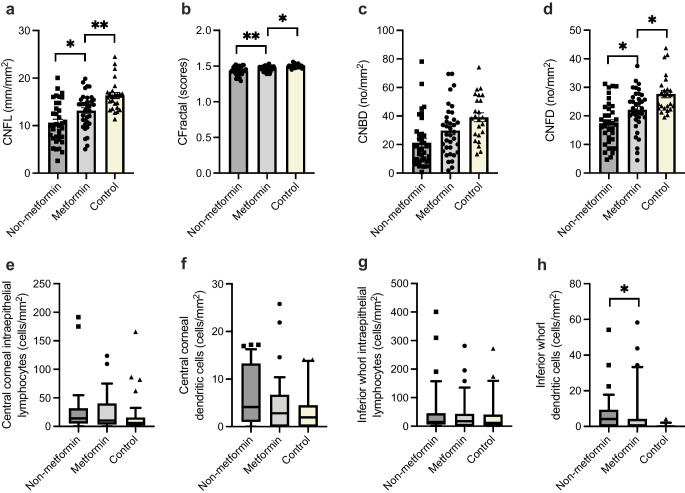
Group comparison of corneal nerve parameters for metformin, non-metformin and control groups. Values are expressed as mean ± SE for (**a**) corneal nerve fibre length (CNFL), (**b**) corneal fractal dimension (CFractal), (**c**) corneal nerve branch density (CNBD) and (**d**) corneal nerve fibre density (CNFD). Values presented as a box and whiskers plot for (**e**) central corneal intraepithelial lymphocytes, (**f**) central corneal dendritic cells, (**g**) inferior whorl intraepithelial lymphocytes, and (**h**) inferior whorl dendritic cells. The central line represents the median and the upper and lower limits of the box represent the 25^th^ and 75^th^ percentiles. The whiskers represent the 10^th^ and 90^th^ percentiles. *p<0.05, **p<0.01

The metformin group demonstrated superior corneal nerve parameters compared with the non-metformin group, with significantly higher corneal nerve fibre density (*p*=0.020), corneal nerve fibre length (*p*=0.020) and corneal fractal dimension (*p*=0.003). All corneal nerve fibre parameters for the metformin group were closer to control values when compared with the non-metformin group (Fig. 1). The changes in corneal nerve parameters were accompanied by a lower inferior whorl dendritic cell population in the metformin group compared with the non-metformin group (*p*=0.043). At the inferior whorl region, dendritic cells were present in 36% of the metformin group, 69% of the non-metformin group and 12% of the healthy control group. There were no significant differences in IEL density between groups. Regression analysis was undertaken using age; sex; duration of disease; HbA_1c_; eGFR; insulin use; dose and duration of metformin therapy in the metformin group; and time since cessation of metformin therapy in the non-metformin group. These factors did not influence corneal neuroimmune parameters between the two groups.

The mean TNS was 4.6±1.0 in the metformin group and 7.1±1.3 in the non-metformin group (*p*=0.175). The metformin group had a non-significant higher sural sensory amplitude (metformin: 9.5±1.2 µV, non-metformin: 7.6±1.2 µV, *p*=0.240) and tibial motor amplitude (metformin: 6.9±0.6 mV, non-metformin: 5.6±0.8 mV, *p*=0.160), compared with the non-metformin group (Table 1).

## Discussion

The current study has shown that metformin-treated individuals demonstrated superior corneal nerve parameters and neuroimmune tone after controlling for significant demographic and metabolic factors, including sex. Sex proportions were not statistically different across treatment and control groups, and regression analysis found that sex did not influence corneal neuroimmune parameters. A limitation of the study is that ~55% of the non-metformin group had received metformin in the past, and had ceased the medication, on average, 5 years previously. Although this study matched groups for all significant demographic and metabolic factors, it is nevertheless limited by its cross-sectional design. It is possible that other factors (e.g. smoking history, exercise levels) may have influenced study outcomes. As this study was conducted at a tertiary referral centre, assessments could not be conducted prior to metformin commencement, as metformin is routinely initiated in the primary care setting as first-line therapy. Future longitudinal studies comparing neuropathy outcomes before and after commencement of metformin therapy are required to confirm our findings. While our previous work found superior neuropathy scores in metformin-treated patients, the present study failed to demonstrate a significant difference in these parameters, likely due to underpowering, as this study was powered to detect changes in corneal morphology, rather than clinical symptoms [9]. Overall, the clinical significance of this study’s findings lies in that IVCCM can detect nerve injury in DPN before the development of neuropathy symptoms or electrophysiological abnormalities [3]. Given that previous work has shown that IVCCM is sensitive to intervention in people with DPN, this study used corneal nerve parameters as a surrogate marker for neuropathy status in people with diabetes.

While our study demonstrated that the metformin group had a lower dendritic cell density compared with the non-metformin group at the most length-dependent region of the sub-basal plexus, it should be noted that there is an inversely proportional relationship between corneal nerve fibre length and dendritic cells [10]. Interestingly, the density of IEL was similar between the treatment groups. Studies have found no consistent difference in IEL density between people with type 2 diabetes and control individuals [11]. This may explain the limited effect of metformin on IEL populations. However, without spatial motility analysis of IEL, this study cannot definitively conclude that there are no changes to corneal lymphocytes with metformin therapy. As metformin's neuroprotective effects have been attributed to its association with ameliorating inflammation [4], the reduced inferior whorl dendritic cell density may reflect a de-escalation of the neuroimmune abnormalities in DPN.

In terms of local corneal effects, studies conflict as to whether metformin exerts a clinically significant effect on corneal endothelial cell density [12, 13], and while animal studies have identified that metformin attenuates keratopathy in animal models [14], the effect of metformin on corneal denervation, epithelial injury and pyroptosis has yet to be explored in humans. In light of our previous work, which demonstrated superior peripheral nerve morphological outcomes with metformin therapy [9], the present study suggests that metformin may protect against corneal denervation through a systemic effect on peripheral nerves in DPN. By comparison, topical administration of insulin has demonstrated efficacy in facilitating wound healing of diabetic neurotrophic keratopathy through the RTK-PI3K/Akt/mTOR axis and Wnt/β-catenin signalling in in vitro and animal studies, respectively [15–17]. Future studies could explore whether systemic or topical metformin protects against the development of diabetic neurotrophic keratopathy and corneal ulcers.

In conclusion, the present study provides further evidence that metformin may be neuroprotective in DPN.

## Data Availability

The data that support the findings of this study are not publicly available due to institutional ethics review board restrictions (Human Research Ethics Committee of the South Eastern Sydney Local Health District HREC Ref. 14/012).

## Abbreviations

DPN: Diabetic peripheral neuropathy
IEL: Intraepithelial lymphocytes
IVCCM: In vivo corneal confocal microscopy
TNS: Total Neuropathy Score
